# Biomarkers of Radiosensitivity in A-Bomb Survivors Pregnant at the Time of Bombings in Hiroshima and Nagasaki

**DOI:** 10.5402/2011/264978

**Published:** 2010-09-29

**Authors:** Edward F. Miles, Yoshimi Tatsukawa, Sachiyo Funamoto, Naoko Kamada, Eiji Nakashima, Yoshiaki Kodama, Thomas Seed, Yoichiro Kusonoki, Kei Nakachi, Saeko Fujiwara, Masazumi Akahoshi, Kazuo Neriishi

**Affiliations:** ^1^Division of Radiation Oncology, Department of Radiology, Naval Medical Center Portsmouth, 620 John Paul Jones Circle, Portsmouth, VA 23314, USA; ^2^Department of Clinical Studies (Hiroshima), Radiation Effects Research Foundation (RERF), 5-2 Hijiyama Park, Minami-ku, Hiroshima City, Japan; ^3^Department of Statistics, RERF, 5-2 Hijiyama Park, Minami-ku, Hiroshima City, Japan; ^4^Department of Genetics, RERF, 5-2 Hijiyama Park, Minami-ku, Hiroshima City, Japan; ^5^Associate Chief of Research, RERF, 5-2 Hijiyama Park, Minami-ku, Hiroshima City, Japan; ^6^Department of Radiation Biology/Molecular Epidemiology, RERF, 5-2 Hijiyama Park, Minami-ku, Hiroshima City, Japan; ^7^Department of Clinical Studies (Nagasaki), RERF, 8-6 Nakagawa 1-chome, Nagasaki City, Japan

## Abstract

*Purpose*. There is evidence in the literature of increased maternal radiosensitivity during pregnancy. *Materials and Methods*. We tested this hypothesis using information from the atomic-bomb survivor cohort, that is, the Adult Health Study database at the Radiation Effects Research Foundation, which contains data from a cohort of women who were pregnant at the time of the bombings of Hiroshima and Nagasaki. Previous evaluation has demonstrated long-term radiation dose-response effects. *Results/Conclusions*. Data on approximately 250 women were available to assess dose-response rates for serum cholesterol, white blood cell count, erythrocyte sedimentation rate, and serum hemoglobin, and on approximately 85 women for stable chromosome aberrations, glycophorin A locus mutations, and naïve CD4 T-cell counts. Although there is no statistically significant evidence of increased radiosensitivity in pregnant women, the increased slope of the linear trend line in the third trimester with respect to stable chromosome aberrations is suggestive of an increased radiosensitivity.

## 1. Introduction

Several studies suggest that maternal radiosensitivity increases during pregnancy, and that this effect is mediated, at least in part, by the elevated levels of steroid and/or nonsteroid maternal hormones during pregnancy [1–11]. The exact biochemical mechanism of this proposed increased radiosensitivity is not well understood.

The Adult Health Study (AHS) population at the Radiation Effects Research Foundation (RERF) includes approximately 20,000 participants exposed to radiation during the bombings of Hiroshima and Nagasaki, Japan in 1945 that have been followed with biennial medical exams. In addition to significant effects on cancer induction, there are a number of long-term dose-response effects detectable in A-bomb survivors [12–17]. A subpopulation of women that were pregnant at the time of the bombing and were exposed to radiation provides a unique opportunity to test the hypothesis that pregnant women show increased radiosensitivity. Our premise was that this increased radiosensitivity during pregnancy might be manifested by a greater dose-response in long-term effects in noncancer parameters. By comparing the difference in dose-response in pregnant women with that in nonpregnant women, a relative measure of radiosensitivity might be derived. 

Our analysis focused on previously evaluated general population dose-response lateeffects of radiation in A-bomb survivors on serum cholesterol (increased) [16], white blood cell (WBC) count (increased) [15], erythrocyte sedimentation rate (ESR) (increased) [15], hemoglobin (HGB) level (decreased) [17], stable chromosome aberration frequency (increased) [12], glycophorin A (GPA) locus mutation rate (increased) [18], and naïve CD4 T-cell counts (decreased) [13]. 

We herein assess whether pregnancy at the time of the bombings increased a woman's radiosensitivity by comparing the long-term effects doseresponses in pregnant women with that of non-pregnant women in the seven parameters noted above. 

## 2. Materials and Methods

### 2.1. Study Parameters

For purposes of analysis, the seven parameters were broadly categorized as “indirect” or “direct” effects. The delineation was loosely based on whether or not the endpoint measured depended on a more “indirect” effect of radiation, that is, requiring a cellular intermediary or sustained cytokine interaction to manifest the end effect (cholesterol, WBC, ESR, HGB) or was the result of a more “direct” effect on a cell-type (stable chromosome aberration, GPA locus mutation, and naïve CD4 T-cell count). Those categories are not strictly defined, but are helpful in interpreting our results.

### 2.2. Study Population

Subject data was obtained from mothers of the 3,631 *in utero* study subjects that were originally registered with the Atomic Bomb Casualty Commission (ABCC)/RERF. A total of 2,452 women that were pregnant at the time of the bombing (ATB) with a known dose were included in the present study. Only 325 of these women participated in some way in any of the AHS data collection cycles. Attrition due to loss from followup and death further diminished the available study population. The type of laboratory study data available increased in later data collection cycles as more sophisticated analytical techniques were developed. 

Due to the disparity in data availability between the “indirect” and “direct” effects, we conducted two separate cross-sectional analyses for this report. The first analysis was performed on data collected during AHS Cycle 2 (1960–1962), which had the maximum participation (250) of women that were pregnant ATB. Laboratory data from this cycle included the “indirect” effect markers (serum cholesterol, WBC, ESR, and HGB). The second analysis involved data from women that participated in AHS Cycle 22 (2000–2002) when “direct” effect test data (chromosome aberration, GPA locus mutation, and naïve CD4 T-cell count) became available. Due to attrition, data from a maximum of only 85 pregnant women was available for analysis in Cycle 22. 

Separate and distinct populations for each of the cross-sectional analyses were also drawn from the AHS database for age and city-matched women that were not pregnant ATB. Since the number of pregnant women was limited, we expanded the age range for non-pregnant women to include as large an amount of women as possible. Pregnant women and non-pregnant women with unknown dose were excluded from either analysis. The final ratio of non-pregnant to pregnant women was approximately 10 : 1 for all study parameters. Demographic information for the entire study cohort is presented in Table 1. Demographic information for the various subgroups is presented in Tables 1 and 2.

### 2.3. Measurement Methods

#### 2.3.1. Total Cholesterol

Nonfasting serum cholesterol levels were measure by the Kendall-Abell method [16].

#### 2.3.2. WBC Count

Anticoagulated blood samples were collected for measurement. WBC counts were obtained manually by the Melangeur method [15]. 

#### 2.3.3. ESR

Anti-coagulated blood samples were collected for measurement. ESR was measured by the Wintrobe method [19] and corrected by a reading of the Wintrobe diagram based on volume of packed red cells. 

#### 2.3.4. HGB Level

Hemoglobin level was measured by a manual procedure with quality control procedures in place to maintain reproducibility and consistency in laboratory results [17].

#### 2.3.5. Stable Chromosome Aberration Frequency

Chromosome spreads from peripheral blood lymphocytes were prepared and Giemsa stained by conventional procedures [20]. They were then classified into seven groups (A to G). Metaphases were photographed for further karyotype analysis if there was any definite or suspected change in the number of chromosomes for any group. Chromosome aberrations were classified into one of three types: reciprocal translocations, pericentric inversions, or small deletions. One hundred cells were scored for each blood sample. The proportion of cells containing at least one aberration per 100 cells was recorded [12].

#### 2.3.6. GPA Locus Mutation Rate

A single-beam cell sorter, FACStar (Becton Dickinson Immunocytometry Systems, San Jose, CA), was used to sort four types of variant erythrocytes lacking the expression of one GPA allele that were distinguished from normal MN heterozygous cells. Those four variants are MΦ, NΦ, MM, and NN. Mutant cells displaying a hemizygous or homozygous phenotype were sorted onto a glass slide, and counted under a fluorescence microscope. Typically, 106 erythrocytes were assayed per sample [14]. Among those four mutant types, the reproducibility of NN cells was low, and MM mutant frequency was significantly affected by overlapping MΦ mutants. Thus, in the report the statistical analysis was undertaken for the mean of MΦ and NΦ hemizygous mutations. 

#### 2.3.7. Naïve CD4 T-Cell Counts

Analytical flow cytometry was conducted in a FACScan machine (BD Biosciences, San Jose, CA). Expression of CD45RA and CD4 molecules was analyzed with FITC-labeled and PE-labeled antibodies, respectively. In every measurement, approximately 20,000 cells were analyzed. The percentage of CD45RA^−^/CD4^+^ (naïve CD4 T-cells) in peripheral lymphocytes was determined [13]. 

### 2.4. Statistical Methods

Separate linear regression analyses were performed based on the indirect and direct outcomes selected. The outcomes for WBC, cholesterol and GPA were logtransformed with base 10 to approximate the transformed outcome as normal distribution. 

For three of the indirect outcomes (HGB, WBC count, and ESR), our model accounted for the influence of city (Hiroshima or Nagasaki), smoking history (current, former, never), age ATB (age at exposure), history of inflammatory disease/inflammatory process (yes or no), cancer history (yes or no), bone marrow radiation dose (DS02) in Gy (continuous variable), and pregnancy (yes or no ATB). Thus, the assumed simple model for the *i*th subject's outcome can be expressed as

(1) $$\begin{array}{cc} E\left(y_{i}\right) & =\beta_{0}+\beta_{1}C_{i}+\beta_{2}\text{S}{\text{m}}_{i}+\beta_{3}\text{A}{\text{a}}_{i}+\beta_{4}\text{I}{\text{D}}_{i}\\ & \;+\beta_{5}{\mathrm{AC}\;}_{i}+\beta_{6}D_{i}+\beta_{7}P_{i}, \end{array}$$

where *β*'s are regression coefficients, *E*(*y*_*i*_) is the expectation for the *i*th subject for the transformed or untransformed outcome *y*_*i*_, *C*_*i*_ is the Nagasaki indicator, Sm_*i*_ is the smoking indicator, Aa_*i*_ is the adjusted age equal to (age ATB-20)/10, ID_*i*_ and AC _*i*_ are inflammatory disease and any cancer indicators, respectively, *D*_*i*_ is the radiation dose, and *P*_*i*_ is the pregnancy indicator. 

For total cholesterol, and the direct outcomes (naïve CD4 T-cell count and GPA mutation rate), the independent variables are city, adjusted age ATB, radiation dose and pregnancy. Thus the assumed model for the *i*th subject for these parameters can be expressed as

(2) $$E\left(y_{i}\right)=\beta_{0}+\beta_{1}C_{i}+\beta_{2}{\text{Aa}}_{i}+\beta_{3}D_{i}+\beta_{4}P_{i},$$

where *β*'s are regression coefficients, *E*(*y*_*i*_) is the expectation for the *i*th subject for transformed or untransformed outcome *y*_*i*_. 

Because of the level of dispersion in the chromosome aberration frequency results, a binomial model with linear probability was used that had city, age ATB, smoking, radiation dose, and pregnancy indicator as independent variables and overdispersion was accounted for. The model for chromosome aberration probability is

(3) $$E\left(y_{i}\right)=\beta_{0}+\beta_{1}C_{i}+\beta_{2}\text{A}{\text{a}}_{i}+\beta_{3}\text{S}{\text{m}}_{i}+\beta_{4}D_{i}+\beta_{5}P_{i},$$

where *β*'s are regression parameters, *E*(*y*_*i*_) is expectation of chromosome aberration rate *y*_*i*_. The Wald test was used to assess significance of the effect. 

## 3. Results

A tabular summary of our findings is presented in Tables 3.

### 3.1. Indirect Studies

No overall dose-response effect was found in serum cholesterol, WBC count, or ESR. Neither was there a statistically significant difference in the slopes of the regression lines for pregnant versus non-pregnant women. There was a suggestion of a statistically significant overall dose-response decrease (*P* = .088) in HGB level of 0.067 g dl^−1^ Gy^−1^. However, the slope of the regression lines for HGB level for pregnant versus non-pregnant women was not statistically different.

### 3.2. Direct Studies

**Figure 1:**
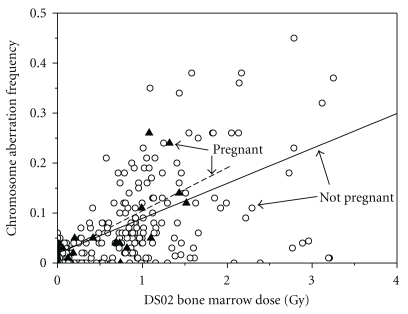
Scatter plot of chromosome aberration frequency in pregnant versus non-pregnant women with linear trend lines for both groups. Open circles reflect control data from non-pregnant women, while solid triangles reflect data from women pregnant ATB.

**Figure 2:**
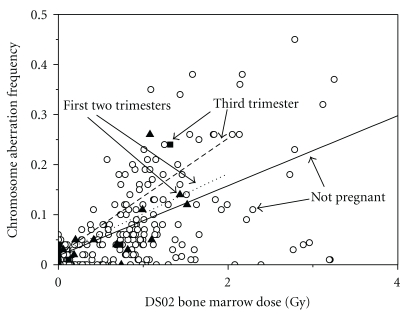
Scatter plot of chromosome aberration frequency in pregnant versus non-pregnant women (first two trimesters separated from third trimesters) with linear trend lines for each group. Open diamonds reflect control data from non-pregnant women, solid triangles reflect women pregnant in the first two trimesters ATB, and solid squares reflect women pregnant in the third trimester ATB.

Chromosome aberration frequency demographic information is presented in Table 2(b). A statistically significant overall dose-response *increase* in stable chromosome aberration frequency of 0.899 Gy^−1^ (*P* < .001) was found. The slope of the regression line for pregnant women in Figure 1 indicated an overall *increase* in stable chromosome aberration frequency (1.672 Gy^−1^), although this line was not statistically different from the line for non-pregnant women (0.878 Gy^−1^), *P* = .378. Although the numbers are very small, for those exposed in the third trimester, the dose-response trend line in Figure 2 is positive and steeper, suggesting a possibility of increased radiosensitivity.

A statistically significant overall dose response increase in GPA locus mutation rate of 26.25 Gy^−1^ (*P* < .001) and a decrease in naïve CD4 T-cell counts of 1.43 Gy^−1^ (*P* = .001) were found. The slope of the respective regression line for pregnant women versus non-pregnant women was not statistically different.

## 4. Discussion

### 4.1. Review of Published Material

Several authors have found that the number of chromosome aberrations in *ex vivo* irradiated peripheral blood lymphocytes (PBL) correlates well with the degree of normal tissue late toxicity after radiation therapy and may be useful as an indicator of a patient's inherent radiosensitivity [21–23]. Alternatively, interphase cells can be examined for the presence of micronuclei that may be used as an indicator of the degree of radiosensitivity [24–27]. More recently, researchers have used gene expression profiles of PBL to predict severe late toxicity with radiation therapy [28]. These studies are in seeming contrast to work by Sposto et al. who concluded that individual differences in the dose response of chromosome aberrations were not significant enough to affect the biodosimetry data in a study of A-bomb survivors [29]. 

There are several preclinical studies that suggest that a biochemical environment that emulates pregnancy may be associated with an increased rate of radiosensitivity, albeit early radiosensitivity as measured primarily by chromosome aberrations in PBL shortly after irradiation. This literature is summarized below. 

Sharma and Das. demonstrated that there was a statistically significant increase in spontaneous sister chromatid exchanges (SCE) in human PBL from females in the third trimester of pregnancy compared to non-pregnant females (10.7 versus 6.5, *P* < .001) [10]. When estrogen, progesterone, and human chorionic gonadotropin were added in physiologic concentrations consistent with pregnancy *in vitro* to PBL from non-pregnant females, a statistically significant increase in SCE was noted after X-ray irradiation to 2 to 3 Gy (9.7 versus 5.6, *P* < .001). 

Roberts et al. demonstrated a statistically significant difference between females and male subjects in the total number of chromosome aberrations (*P* = .006) and a trend towards significance in dicentric formation (*P* = .07) in PBL after exposure of whole blood to high dose-rate radiation (1.75 Gy at 0.29 Gy/min) [9]. The range of sensitivity in the group of 50 healthy female volunteers was also significantly greater than in the male volunteers. Interestingly, there was also a large variation in sensitivity over time among samples from the same volunteer, implying a transitory effect. In evaluating this variation, it was noted that two subjects had started taking hormonal therapy during the course of sample collection. One volunteer, who was taking conjugated estrogen alone, had no increase in chromosomal aberrations over the course of the study. A second volunteer, taking a progesterone/estrogen combination demonstrated a 68% increase in her chromosome aberration count after starting on hormonal therapy and this level continued after termination of the hormone therapy. 

Building on previous work showing similar results in a pregnant mouse model [5, 6], Ricoul et al. demonstrated that the rate of radiation-induced chromosomal breaks in *ex-vivo* irradiated PBLs was, on average, higher in pregnant versus non-pregnant humans [8]. Due to a much lower variation in fetal chromosomal aberration with the term of pregnancy in their previous work with mice, the authors suggested a relationship with maternal hormones as these fluctuations are more pronounced on the maternal side during the course of pregnancy. In a small longitudinal portion of this study, radiation-induced chromosome aberrations in PBL were noted to increase in the 30th week of gestation and drop markedly after delivery of term infants and that this increased rate was more closely correlated with the serum level of progesterone than estrogen. 

Ricoul et al. subsequently demonstrated that the *in vitro* addition of progesterone prior to irradiation resulted in an increased frequency of chromosome rearrangements and that this effect was particularly efficient at the G1/S transition period. They suggest that the progesterone may stimulate accelerated, and hence more error-prone, chromosome repair resulting in survival past S-phase, albeit with illegitimate chromosome rearrangements [7]. 

Kanda and Hayata investigated the effect of incubation of human PBL with various levels of estradiol during and after irradiation to 3 Gy at 1.4 Gy/min [3]. They found that at a level of 100 ng/ml (consistent with the third trimester of pregnancy) there was a statistically significant 20% increase in the radiation-induced formation of dicentrics and centric rings (*P* < .05), and that this level is in the range associated with chromosome aberrations found in irradiated lymphocytes from patients with hereditary retinoblastoma [4]. They speculate based on other authors work with similar findings in peripheral blood mononuclear cells [2], that it is possible that the estradiol reduced the rate of apoptosis resulting in a higher frequency of chromosomal aberrations at subsequent metaphase.

Baeyens et al. found higher levels of micronuclei in an *in vitro* assay of irradiated PBL from pregnant (third trimester) versus non-pregnant humans [1]. They suggest that weak variations in hormonal levels fail to enhance PBL radiosensitivity, but that important increases in serum estradiol and progesterone, such as those found during pregnancy, could increase PBL radiosensitivity.

Similar to the speculation by Kanda et al. noted above, Vares et al. investigated the role of progesterone in modulating the effects of radiation on several human breast cancer cell lines [11]. They found that in progesterone receptor positive cell lines, a 10 nM concentration of MPA (a synthetic progestin) was sufficient to significantly inhibit apoptosis induced by *in vitro* irradiation of 4 Gy. Progesterone treatment was also shown to counterbalance radiation-induced growth arrest and consequently increase the frequency of chromosomal aberrations (measured via micronucleus assay) in subsequent cell cultures.

In summary, several researchers have found that *in vitro* irradiation of PBL from women that were pregnant, or PBL irradiated in media that emulates concentrations of steroid hormones consistent with pregnancy, tend to have a higher level of chromosome aberrations suggestive of an increased radiosensitivity. 

In conducting this retrospective analysis, we expected that the radiation dose-response in pregnant women, as indicated by the slope of the individual regression line, for our selected parameters would be steeper than that found in the group of non-pregnant female A-bomb survivors.

It should be noted that there is a potential for significant selection bias at several levels in our analysis. First, as noted in the methods section, only a small fraction of the women that were pregnant at the time of bombings participated in the AHS, and not all of these women had appropriate data available for analysis. In addition, the selection of women to participate in the original chromosome aberration and GPA locus mutation rates published by previous authors was predicated on a higher radiation exposure dose so extrapolation of the dose rate effect to the lower doses experienced by the women in the present study may be problematic.

### 4.2. Expected Results and Present Study Findings:Indirect Studies

Wong et al. reported a significant dose-dependent increase in serum cholesterol level in a study involving more than 9,800 A-bomb survivors with a mean dose of 1.09 Sv [16]. Neriishi et al. reported a statistically significant dose-response increase in WBC count (71.0 cells mm^−3^ Gy^−1^, *P* = .015) and an increase in ESR (1.58 mm hr^−1^ Gy^−1^, *P* = .0001) among more than 6,000 A-bomb survivors with a mean dose of 0.38 Gy [15]. Wong et al. demonstrated a statistically significant reduction in serum hemoglobin level for those exposed to 1 Gy bone marrow dose of 0.10 g/dl (95% CI 0.04–0.16) at 40 years of age and 0.24 g/dl (95% CI 0.08–0.40) at 80 years of age in a study of more than 7,000 survivors with a median dose of 0.43 Gy [17]. While our findings were not inconsistent with those of these previous studies, overall, there is no indication of increased radiosensitivity in pregnant women with respect to serum cholesterol, WBC count, serum ESR, or serum hemoglobin.

These results are based on data collected during Cycle 2 of the AHS, 13–15 years after the bombings, and may not reflect the true long-term incidence of late effects in these parameters. The published dose-response effects to date have involved the study of responses over much longer time intervals. However, Cycle 2 contained the largest number of women that were pregnant at the time of the bombings, so using a later cycle for analysis would further limit the statistical power of any findings. 

### 4.3. Expected Results and Present Study Findings:Direct Studies

In a study involving over 3,000 A-bomb survivors, Kodama et al. demonstrated the existence of stable chromosome aberrations (at least one translocation or inversion per 100 lymphocytes per person) occurred at a rate related to the radiation exposure dose with an alpha/beta ratio of approximately 1.7 Sv (95% CI 0.9–4) [12]. 

For our study, an overall positive dose-response chromosome aberration effect was found that is in accordance with that found by Kodama et al. Although their results are not statistically different, both pregnant and non-pregnant women show a significant positive dose-response effect. Graphically in Figure 1, this appears to be more substantial in pregnant women, but due to overall low data availability, statistical significance was not attained. As can be seen in Table 2(b), the pregnant women cohort represents a statistically older population that was subject to statistically suggestive lower median radiation dose. Overall, there is no clear indication of increased radiosensitivity in pregnant women with respect to chromosome aberration frequency. 

As shown in Figure 2, the dose-response trend line is suggestively steeper for the small number of women in the third trimester. However, power calculations demonstrate that data from an additional 684 pregnant women in the third trimester would be required to show a statistically significant difference from non-pregnant women. Additional chromosome aberration data is being collected using more modern techniques, but there were simply not enough pregnant women in the third trimester to allow for statistically significant separation of the dose-response trend lines.

Regarding GPA locus mutation, Kyoizumi et al. studied the mutation rate among more than 1,000 A-bomb survivors with a mean age of 64 and found a doubling dose of 1.2 Sv (95% CI 0.95–1.56) with a minimum dose to detect increased mutation of 0.24 Sv (95% CI 0.041–0.51) [14]. It was postulated that the mutability of the GPA locus is indicative of the nonspecific mutability of all somatic cell lines, including genetic loci of cancer-associated genes [18].

An overall positive dose-response effect with respect to GPA locus mutation was found that is in accordance with that found by Kodama et al.; the overall dose-response rate was 26 mutations Gy^−1^. However, there was no significant difference in the regression lines for pregnant versus non-pregnant women. 

Regarding naïve CD4 T-cell counts and building on previous work that demonstrated a decreased count in 159 A-bomb survivors exposed to more than 1.5 Gy of radiation [30], Kusunoki et al. studied lymphocytes from 553 survivors and demonstrated a statistically significant dose-dependent decrease in percentage of circulating naïve CD4 and CD8 T-cells. They also studied lymphocytes from 723 atomic bomb survivors and demonstrated a statistically significant dose-dependent decrease in the percentage of circulating naïve CD4 T-cells [13]. 

An overall negative dose-response effect was found that is in accordance with that found by Kusonoki et al.; the overall dose-response rate was a decrease of 1.42 Gy^−1^. However, there was no significant difference in the regression lines for pregnant versus non-pregnant women. 

Numeric result data for all parameters is presented in Table 3.

### 4.4. Rationale for Continued Study

In general, diagnostic imaging and radiation therapy are proscribed during pregnancy, more for the well-documented potential for harm to the developing fetus than for the protection of the expectant mother. However, there can be inadvertent radiation exposure for diagnostic imaging during an occult pregnancy. Also, diagnostic images are occasionally obtained in women during the third trimester since by that time the fetus is generally thought to be less susceptible to catastrophic radiation damage (intrauterine death, microcephaly, mental retardation) and the low-rate of long-term cancer induction risk to the fetus may be outweighed by the need for diagnostic imaging or treatment in the mother [31]. Unfortunately, in previous studies with peripheral blood lymphocytes (PBL), the third trimester appears to be potentially the most at-risk phase for the pregnant women in terms of the potential for increased radiosensitivity [1–11]. For informed consent purposes, physicians must fully relate the potential for increased risk due to the diagnostic or therapeutic maneuver both to the developing fetus as well as the expectant mother. 

What we learn from studies on radiosensitivity and hormones may have implications for understanding the role of the hormonal milieu on radiation therapy-induced tumor and normal tissue effects. Radiation therapy is commonly used in women who may benefit from antiestrogens and aromatase inhibitors as part of their treatment for breast cancer. It is important to understand if these agents, which affect the production or interaction of estrogen with its cellular receptor, markedly alter the sensitivity of the tumor and/or normal tissues to the effects of radiation therapy. Small studies have demonstrated in vitro cell-cycle redistribution effects and alterations to tumor cell survival curves with concurrent anti-estrogen exposure [32, 33]. 

Clinically, several retrospective studies have demonstrated no increased acute or late adverse effects or impact on local control with concurrent versus sequential radiation therapy and hormonal therapy for early stage breast cancer [34–36], but several other studies demonstrate increased incidence of early and late lung injury [37] as well as increased breast fibrosis [38]. An *in vivo* study with a human breast cancer cell line demonstrated a radiosensitizing effect of a therapeutic level of letrozole with radiation doses ranging from 0 to 4 Gy [39]. 

To date, there are no published *in vivo*, prospective, randomized trials to evaluate the concurrent use of anti-estrogens or aromatase inhibitors and radiation therapy. 

It is also possible that other metabolic conditions other than the level of circulating hormones may also affect the relative radiosensitivity of women who are pregnant at the time of exposure. For example, as pregnancy is a hypermetabolic state and is associated with increased oxidative stress [40, 41], it is possible that this higher metabolic state results in the circulation of an increased level of free radicals that act to exacerbate the oxidative damage of radiation exposure.

## 5. Conclusions

There is no statistically significant evidence of changes in various biomarkers to suggest an increased radiosensitivity in women that were pregnant ATB in Hiroshima and Nagasaki as compared to non-pregnant control subjects. We compared the late effects of changes in serum cholesterol, WBC count, ESR, HGB, stable chromosome aberration, GPA locus mutations, or naïve CD4 T-cell counts. 

The increased slope of the trend line in the third trimester with respect to stable chromosome aberrations is suggestive of an increased radiosensitivity, but there are not enough pregnant women in the third trimester in the AHS database to demonstrate statistical significance.

A separate protocol is underway to determine if cancer incidence (particularly breast, skin and thyroid), cancer mortality, or all-cause mortality is increased in women that were pregnant ATB. This study will have the advantage of using the much larger Life Span Study (LSS) database, containing information on approximately 120,000 survivors, and may have sufficient power to identify even small differences. 

The relative efficacy and toxicity associated with the concurrent use of anti-estrogens and aromatase inhibitors should be evaluated in a randomized trial setting with long-term followup to determine if these agents change the therapeutic index by altering the production and/or cellular interaction of estrogen.

## Figures and Tables

**Table 1:** Distribution of pregnant and non-pregnant women in A-bomb survivors by cycle, city, and age at the bombs.

Cycle	City	Pregnant	Age ATB					
			<20	<25	<30	<35	35+	Total
2	Hiro	No	326	480	368	389	1264	2827
		Yes	2	50	35	33	38	158
	Naga	No	167	186	127	85	211	776
		Yes	4	10	17	5	9	45

22	Hiro	No	217	319	145	99	45	825
		Yes	3	32	18	7	1	61
	Naga	No	139	129	64	31	13	376
		Yes	3	6	10	2	0	21

H: Hiroshima; N: Nagasaki.

**Table:** (a)

Items		Pregnant	Not pregnant	Two sided
Cholesterol				
	Number	58	819	
	Median age ATB (range)	27 (17, 43)	31 (17, 47)	0.090
	First trimester (%)	22 (37.9)		
	Second trimester (%)	26 (44.8)		
	Third trimester (%)	10 (17.2)		
	Hiroshima (%)	36 (62.1)	577 (70.5)	0.231
	Nagasaki (%)	22 (37.9)	242 (29.5)	
	Median dose (mGy) ((range)	172.0 (0, 2862)	115.5 (0, 3380)	0.170
WBC				
	Number	166	2368	
	Median age ATB (range)	28 (17, 44)	30 (17, 47)	0.019
	First trimester (%)	58 (34.9)		
	Second trimester (%)	67 (40.4)		
	Third trimester (%)	41 (24.7)		
	Current Smoker (%)	24 (14.5)	403 (17.0)	>0.5
	Former (%)	3 (1.8)	56 (2.4)	
	Never (%)	139 (83.7)	1909 (80.6)	
	History of Inflammatory of disease (%)	40 (24.1)	643 (27.2)	0.443
	Cancer (%)	1 (0.6)	12 (0.5)	0.693
	Hiroshima (%)	129 (77.7)	1862 (78.6)	0.856
	Nagasaki (%)	37 (22.3)	506 (21.4)	
	Median dose (mGy) (range)	97.2 (0, 2862)	134.6 (33.325)	0.223
ESR				
	Number	101	1466	
	Median age ATB, range	28 (19, 43)	32 (19, 47)	0.001
	First trimester (%)	35 (34.7)		
	Second trimester (%)	41 (40.6)		
	Third trimester (%)	25 (24.8)		
	Current Smoker (%)	14 (13.9)	249 (17.0)	0.206
	Former (%)	0 (0.0)	33 (2.3)	
	Never (%)	87 (86.1)	1184 (0.808)	
	History of Inflammatory of disease (%)	17 (0.168)	340 (23.2)	0.177
	Cancer (%)	1 (1.0)	11 (0.8)	0.747
	Hiroshima (%)	101 (1.0)	1466 (1.0)	< 0.001
	Nagasaki (%)	0 (0.0)	0 (0.0)	
	Median dose (mGy) (range)	76.3 (0, 2391)	157.4 (0, 3278)	0.033

**Table:** (b)

HGB				
	Number	166	2368	
	Median age ATB, range	28 (17, 44)	30 (17, 47)	0.019
	First trimester (%)	58 (34.9)		
	Second trimester (%)	67 (40.4)		
	Third trimester (%)	41 (24.7)		
	Current Smoker (%)	24 (14.5)	403 (17.0)	0.607
	Former (%)	3 (1.8)	56 (2.4)	
	Never (%)	139 (83.7)	1909 (80.6)	
	History of Inflammatory of disease (%)	40 (24.1)	643 (27.2)	0.443
	Cancer (%)	1 (0.6)	12 (0.5)	0.693
	Hiroshima (%)	129 (77.7)	1862 (78.6)	0.856
	Nagasaki (%)	37 (22.3)	506 (21.4)	
	Median dose (mGy) (range)	97.2 (0, 2862)	134.6 (0, 3325)	0.223
Stable chromosome aberration frequency				
	Number	27	313	
	Median age ATB (range)	23 (20,34)	21 (17, 33)	0.003
	First trimester (%)	10 (37.0)		
	Second trimester (%)	11 (40.7)		
	Third trimester (%)	6 (22.2)		
	Hiroshima (%)	18 (66.7)	213 (68.1)	0.947
	Nagasaki (%)	9 (33.3)	100 (32.0)	
	Median Dose (mGy) (range)	121.7 (0, 1518)	287.6 (0, 3252)	0.328
GPA locus mutation				
	Number	26	327	
	Median age ATB (range)	24 (18, 32)	21 (17, 38)	0.005
	First trimester (%)	7 (26.9)		
	Second trimester (%)	11 (42.3)		
	Third trimester (%)	8 (30.8)		
	Hiroshima (%)	23 (88.5)	214 (65.4)	0.029
	Nagasaki (%)	3 (11.5)	113 (34.6)	
	Median Dose (mGy), range	4.3 (0, 1621.4)	80.1 (0, 2977)	0.322
Naïve CD4 T cell count				
	Number	75	887	
	Median age ATB (range)	24 (17, 38)	21 (17, 38)	<0.001
	First trimester (%)	26 (34.7)		
	Second trimester (%)	33 (44.0)		
	Third trimester (%)	16 (21.3)		
	Hiroshima (%)	57 (76.0)	606 (68.3)	0.211
	Nagasaki (%)	18 (24.0)	281 (31.7)	
	Median Dose (mGy), range	66.9 (0, 2027)	81.9 (0, 3311)	0.554

**Table:** (a)

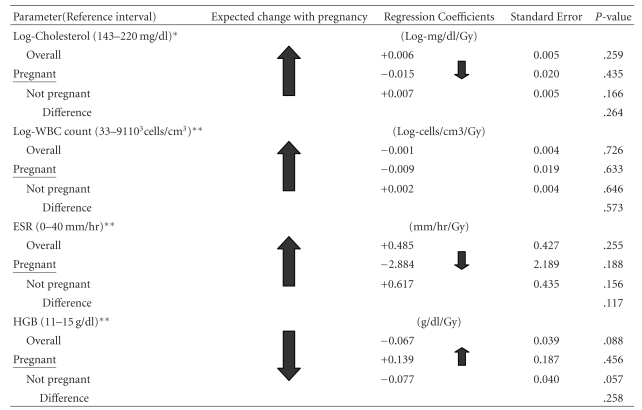

*Adjusted for age and city.

**Adjusted for age, city, smoking, inflammatory disease, and any cancer.

**Table:** (b)

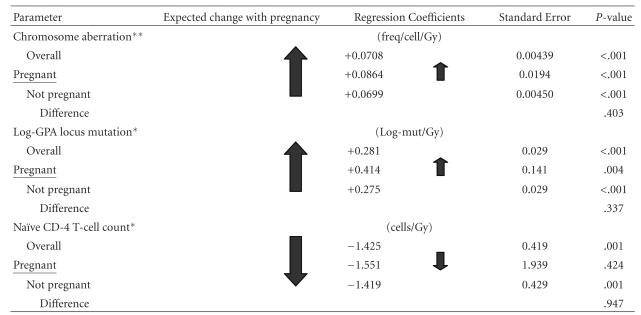

*Adjusted for age and city.

**Adjusted for age, city, and smoking.
